# A factor XIa-activatable hirudin-albumin fusion protein reduces thrombosis in mice without promoting blood loss

**DOI:** 10.1186/s12896-018-0431-4

**Published:** 2018-04-05

**Authors:** William P. Sheffield, Louise J. Eltringham-Smith, Varsha Bhakta

**Affiliations:** 1 Department of Pathology and Molecular Medicine, McMaster University, 1280 Main Street West, Hamilton, ON L8S 4K1 Canada; 2 Centre for Innovation, Canadian Blood Services, Hamilton, ON Canada

**Keywords:** Hirudin, Albumin, Thrombin, Factor XI, Thrombosis, Hemorrhage

## Abstract

**Background:**

Hirudin is a potent thrombin inhibitor but its antithrombotic properties are offset by bleeding side-effects. Because hirudin’s N-terminus must engage thrombin’s active site for effective inhibition, fusing a cleavable peptide at this site may improve hirudin’s risk/benefit ratio as a therapeutic agent. Previously we engineered a plasmin cleavage site (C) between human serum albumin (HSA) and hirudin variant 3 (HV3) in fusion protein HSACHV3. Because coagulation factor XI (FXI) is more involved in thrombosis than hemostasis, we hypothesized that making HV3 activity FXIa-dependent would also improve HV3’s potential therapeutic profile. We combined albumin fusion for half-life extension of hirudin with positioning of an FXIa cleavage site N-terminal to HV3, and assessed in vitro and in vivo properties of this novel protein.

**Results:**

FXIa cleavage site EPR was employed. Fusion protein EPR-HV3HSA but not HSAEPR-HV3 was activated by FXIa in vitro. FVIIa, FXa, FXIIa, or plasmin failed to activate EPR-HV3HSA. FXIa-cleavable EPR-HV3HSA reduced the time to occlusion of ferric chloride-treated murine arteries and reduced fibrin deposition in murine endotoxemia; noncleavable mycHV3HSA was without effect. EPR-HV3HSA elicited less blood loss than constitutively active HV3HSA in murine liver laceration or tail transection but extended bleeding time to the same extent. EPR-HV3HSA was partially activated in citrated human or murine plasma to a greater extent than HSACHV3.

**Conclusions:**

Releasing the N-terminal block to HV3 activity using FXIa was an effective way to limit hirudin’s bleeding side-effects, but plasma instability of the exposed EPR blocking peptide rendered it less useful than previously described plasmin-activatable HSACHV3.

## Background

Nature exploits specific proteolytic cleavage as a mechanism to transform inactive precursor proteins into active enzymes. Numerous examples of this exquisite biological strategy can be found, for instance in: the enzymes of the intrinsic and extrinsic coagulation pathways [1]; the fibrinolytic precursor-enzyme combination of plasminogen and plasmin [2]; and in the proteins of the complement and caspase cascades [3, 4]. Enzyme activation by specific proteolytic cleavage allows for tight regulation of the timing, location, and extent of enzymatic activity. From a biotechnological perspective, attempts have been made to emulate this approach, to produce novel proteins with potential therapeutic applications. Dawson et al. mutated the unique activation site of plasminogen, ordinarily cleaved by tissue-type (tPA) or urokinase-type plasminogen activators, to the thrombin recognition site in coagulation factor XI (FXI) [5]. This change accelerated plasmin formation at the site of pathological blood clots, and the engineered plasminogen was shown to be a superior thrombolytic agent to tPA in dog or rabbit thrombosis models [6]. Other examples of conceptually related approaches include single chain variable fragment (ScFv) antibody-interleukin 12 (IL-12) chimeric proteins in which the ScFv component blocks biological activity of IL-12 until tumour-enriched proteases release IL-12 [7], and coagulation factor IX- (FIX-) albumin fusion proteins that circulate together until FIX is activated and released from albumin via a repetition of its natural activation sites [8].

Hirudin is a small protein found in medicinal leech secretions that potently inhibits the key coagulation enzyme thrombin [9]. Early in the biotechnological production of recombinant hirudin, it was noted that much of its activity was lost if its N-terminus was blocked, even by small peptides [10, 11]. This observation was supported and rationalized by the co-crystallization of hirudin with thrombin, which showed the N-terminus of the small protein inserted into the active site canyon of the enzyme [12]. These findings prompted efforts to engineer a protease-activated “switch” into hirudin, to create a specific way of activating the thrombin inhibitor. Peter et al. fused an anti-fibrin-ScFv to hirudin, separating the fusion protein components with a factor Xa (FXa) cleavage site [13]. Our laboratory fused human serum albumin (HSA) to hirudin, separating the components with a plasmin cleavage site [14]. Zhang et al. capped the N-terminus of hirudin with small peptides cleavable by thrombin, FXa, or FXIa [15].

There is considerable current interest in FXI(a) as a target for antithrombotic therapy [16]. Existing antithrombotic agents come with bleeding risks. Both mice and human patients deficient in FXI are protected from thrombosis, and do not appear to suffer a strong bleeding tendency [17]. Pharmacological down-regulation of FXI mRNA by specific antisense oligonucleotides has been shown to be superior to an existing antithrombotic agent in a phase II clinical trial [18]. For these reasons, in this study we returned to the concept of an activatable hirudin-albumin fusion protein, substituting an FXIa cleavage site N-terminal to the hirudin moiety, and positioning HSA either C-terminal or N-terminal to the FXIa-removable hirudin activity blocking group. We tested the hypothesis that FXIa-activatable hirudin-albumin fusion protein would diminish thrombosis in vivo without promoting bleeding, while maintaining a circulating reserve of latent protein due to the slowly cleared phenotype of HSA. In this study, we found that FXIa-activatable hirudin-albumin had a superior benefit/risk profile to constitutively active hirudin-albumin, but one inferior to our previously reported plasmin-activatable counterpart [14, 19].

## Methods

### Construction of expression plasmids and transformation of *Pichia pastoris*

**Fig. 1:**
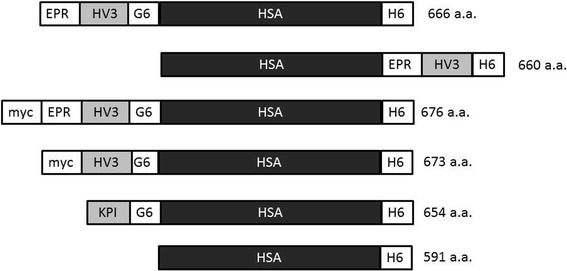
Schematic representation of recombinant proteins employed in this study. All polypeptides contained human serum albumin (HSA) sequences, were expressed in *Pichia pastoris,* and were purified from media conditioned by methanol-induced cultures. Polypeptides are shown in linear format, amino- to carboxyl-terminus, to emphasize their modular design. Protein segments are shown in black boxes (HSA, 585 amino acids), while small proteins (HV3, hirudin variant 3, 66 amino acids, or KPI, the Kunitz Protease Inhibitor domain of protease nexin 2, 57 amino acids) are shown in grey, and peptides (G6, hexaglycine spacer, H6, hexahistidine affinity tag, EPR, FXIa-cleavable tripeptide (i.e. Glu-Pro-Arg, and myc, myc oncoprotein epitope tag, EQKLISEEDL) in white. From top to bottom, the following recombinant, His-tagged proteins are depicted, with their length in amino acids ([a.a.], at right of linear depiction): EPR-HV3HSA; HSA-EPRHV3; mycEPR-HV3HSA; mycHV3HSA; KPIHSA; and HSA

Oligonucleotides were obtained through the Institute for Molecular Biology and Biotechnology (MOBIX) at McMaster University (Hamilton, ON). DNA sequencing to confirm the coding sequences of all plasmids was performed by the same facility. Three of four novel expression plasmids were constructed as follows. Plasmid pPICZ9ssHV3HSAH_6_ [20, 21] was DNA-amplified using sense and antisense paired oligodeoxyribonucleotide primers in reactions catalysed by heat-stable Phusion DNA polymerase (Thermo Fisher Scientific, Waltham, MA, USA). Primer sequences for each construct are given in Table 1. Following PCR, products were restricted with XhoI and XbaI, gel-purified, and ligated to the 5237 bp XhoI-XbaI double digestion fragment of pPICZ9ssHV3HSAH_6_. Following transformation of competent *E. coli* DH5α subclones with appropriate restriction profiles were verified by DNA sequencing. Clones of correct sequence were designated pPICZ9ss(protein), where (protein) was either EPR-HV3HSA, mycEPR-HV3HSA, or mycHV3HSA, as they were designed to direct the synthesis and secretion of these proteins (see Fig. 1). Note that EPR is tripeptide Glu-Pro-Arg in single letter amino acid code, and that myc is EQKLISEEDL in the same notation. The fourth novel expression plasmid, pPICZ9ssHSAEPR-HV3, was constructed in a similar manner, except that the template was pPICZ9ssHSACHV3H_6_ [14], and the PCR product produced using the specific primer pair in Table 1 was restricted with SnaBI and XbaI and ligated to the major restriction endonuclease digestion product of pPICZ9ssHSACHV3H_6_ [14] to yield pPICZ9ssHSAEPR-HV3. Each sequence-verified plasmid described above was linearized via SacI digestion and was then used to transform *Pichia pastoris* strain X33 to Zeocin (Invitrogen) resistance as previously described [14, 19–21].

**Table 1:** Oligonucleotide sequences. Oligonucleotide primers used in expression plasmid construction. The unique order number, short name, DNA sequence, and purpose is shown. Sense EPR, myc, and mycEPR oligonucleotides were separately paired with antisense myc/EPR oligonucleotide for amplification and restriction with XhoI and XbaI. HSA sense and antisense clEPRHV3 were paired for amplification and restriction with SnaBI and XbaI. Additional details are provided in the Materials and Methods

Number	Name	Sequence	Purpose
123,620,623	Sense EPR	5’-TCTCTCGAG AAAAGAGAGC CTAGAATCAC CTACACAGAC TGC-3’	Mutate and amplify codons to alter HV3HSA cDNA to EPR-HV3HSA, provide XhoI site
123,620,624	Sense myc	5’-TCTCTCGAGA AAAGAGAACA AAAACTCATC TCAGAAGAGG ATCTGATCAC CTACACAGAC TGC-3′	Mutate and amplify codons to alter HV3HSA cDNA to myc-HV3HSA, provide XhoI site
123,620,625	Sense mycEPR	5’-TCT CTC GAG AAA AGA GAA CAA AAA CTC ATC TCA GAA GAG GAT CTG GAG CCT AGA ATC ACC TAC ACA GAC TGC-3′	Mutate and amplify codons to alter HV3HSA cDNA to myc-EPRHV3HSA, provide XhoI site
ML-08-6225	Antisense myc/EPR	5′- CTCTCCAAGC TGCTCGAAAA GCTC-3’	Anchor 3′ end of amplification in all 3 cases above, allow inclusion of XbaI site in amplicon
890,140,002	HSA sense	5’-AGGCATCCTGA TTACTCTGTC GTGCTGCTG-3’	Anchor 5′ end of amplification for HSAEPR-HV3 construct, allow inclusion of XbaI site in amplicon
161,722,273	Antisense clEPRHV3	5’ TGTGTACGTAA TTCTAGGCTC GGATCCTAAG GCAGC-3’	Mutate and amplify codons specifying EPR cleavage site for HSAEPR-HV3 construct, allow inclusion of SnaBI site in amplicon

### Expression and purification of albumin fusion proteins

Clonal *Pichia pastoris* cell lines transformed with expression plasmids described above, or pPICZ9ssHSACHV3, pPICZ9ssHSAH_6_ [14], or pPICZ9ssKPIHSA [22], were used to condition buffered minimal medium supplemented with methanol for 72 h. Following neutralization, clarification and ultrafiltration, albumin fusion proteins were purified from concentrated conditioned media by nickel-chelate affinity chromatography as previously described [14, 21].

### Prothrombin time assays

Prothrombin times (PT) were determined using an STA 4 coagulation analyzer (Diagnostica Stago, Asnières-sur-Seine, France). Citrated normal human pooled plasma ([NHPP], Precision Biologic, Dartmouth, NS, Canada) or NHPP immunodepleted of Factors VIII, IX, XI, or XII (Haematologic Technologies, Essex Junction, VT, USA) was combined with purified fusion proteins (45:5 μl by volume) in saline, or saline alone, and incubated at 37 °C for 2 – 240 min prior to initiation of PT measurement through addition of 50 μl Thromborel S PT reagent containing human placental thromboplastin and calcium chloride (Siemens Healthcare Diagnostics, Oakville, ON, Canada). In some experiments, citrated CD-1 mouse pooled plasma was substituted for NHPP.

### Two-stage activation and thrombin activity assays

To determine if albumin fusion proteins possessed latent antithrombin activity, a two-stage assay was employed as previously described [14, 19]. Briefly, albumin fusion proteins (400 nM) were combined with 300 nM purified protease in PPNE buffer (20 mM sodium phosphate, pH 7.4, 0.1% polyethylene glycol 8000, 100 mM NaCl, 0.1 mM EDTA) for varying times. Purified proteases included FXIa, Factor Xa (FXa), Factor XIIa (FXIIa), plasmin (Enzyme Research Laboratories (South Bend, IN, USA)) and recombinant FVIIa (Niastase, Novo Nordisk Canada, Mississauga, ON, Canada). First-stage reaction products were combined 1:1 with 10 nM thrombin in PPNE for 1 min, and then diluted 10-fold into 100 μM S2238 chromogenic substrate (Chromogenix, Lexington, MA, USA). Colour generation from the amidolysis reaction was followed for 5 min to determine the reaction velocity. In some reactions HEPES-buffered saline (HBS; 25 mM HEPES, pH 7.4, 100 mM NaCl) supplemented with 5 mM calcium chloride was substituted for PPNE.

### Electrophoresis and immunoblotting

SDS polyacrylamide gel electrophoresis was carried out under denaturing and reducing conditions using a Mini-PROTEAN III electrophoresis system following the manufacturer’s instructions (BioRad Laboratories, Mississauga, ON, Canada). Replica gels were transferred to nitrocellulose using an iBlot Gel Transfer Device as specified by the manufacturer (Invitrogen, Carlsbad, CA, USA). Immunoblotting was as previously described, using Tris-buffered saline/0.02% Tween 20 (TBST) for washing and 3% BSA in TBST for antibody incubations. Primary antibodies were all monoclonal: mouse anti-HSA (Genway Biotech, San Diego, CA; mouse anti-polyhistidine (Sigma-Aldrich, Oakville, ON, Canada); and mouse anti-myc (Thermo Fisher Scientific, Mississauga, ON, Canada). The secondary antibody was goat anti-mouse IgG, alkaline phosphatase- (AP-) conjugated. Blots were visualized through colour development resulting from the action of AP on substrate nitro blue tetrazolium chloride/5-bromo-4-chloro-3-indolyl-phosphate, toluidine salt (NBT/BCIP).

### Murine carotid artery thrombosis model

The time to occlusion (TTO) of the ferric-chloride treated carotid artery was assessed by Doppler ultrasound as previously described [14, 23–26]. Briefly, CD-1 mice were anesthetized, the carotid artery was exposed surgically, and thrombosis was induced by topical application of a 1 mm^2^ patch of Whatman paper soaked in 10% (*w*/*v*ol) ferric chloride for 3 min. Two min prior to patch application, purified EPR-HV3HSA or mycHV3HSA in saline, or saline vehicle, were injected intravenously at 120 or 40 mg/kg body weight. Arterial blood flow was monitored after patch removal and the TTO defined as the time until flow dropped below 0.1 ml/min. Vessels not occluded at the end of the 60 min observation period were scored with a TTO of 60 min. These and all other experiments with mice were carried out under the terms of an Animal Utilization Protocol (AUP) approved by the Animal Research Ethics Board of the Faculty of Health Sciences of McMaster University. At the close of all experiments, anesthetized mice were euthanized by cervical dislocation as stipulated in the approved AUP.

### Murine LPS/L-NAME fibrin deposition thrombosis model

The deposition of fibrin in the heart and kidney of CD-1 mice treated with lipopolysaccharide (LPS, serotype 0127:B8, Sigma-Aldrich) and vasoconstrictor nitro-L-arginine methyl ester (L-NAME, Alexis, Nottingham, UK) [27, 28]was assessed using radiological methods as previously described [23]. Briefly, L-NAME was given intraperitoneally (IP) (50 mg/kg body weight) at 0, 30, 120, and 240 min; the second injection also contained 2 mg/kg body weight LPS. One minute prior to the first IP injection, albumin fusion proteins in saline or saline vehicle were given at 40 mg/kg body weight with 30 million cpm ^125^I-human fibrinogen labeled using the Iodogen method [29]. Five hours after the first IP injection, mice were euthanized, and hearts and kidneys were excised, washed in ice-cold saline, and organ-associated radioactivity was determined by γ-counting.

### Murine bleeding models

Blood loss subsequent to injury was determined in two previously described models: liver laceration [19]; and tail transection model [14]. In the first model, CD-1 mice were anesthetized and a standard, 5 mm perforating incision was made through an exteriorized liver lobe placed on a tared weigh boat. Shed blood was captured in the weigh boat and combined with shed blood clotted on the lobe surface 15 min after injury. Albumin fusion proteins or saline vehicle were injected intravenously (IV) two minutes prior to injury. In the second model, anesthetized CD-1 mice were also injected IV with albumin fusion proteins or saline vehicle just prior to injury, but the injury in this case was tail transection at a point 1.0 cm from the tip. Shed blood was collected on Whatman paper for 15 min, and subsequently eluted in water. Blood loss was quantified by determining the optical density of the hemolysate at 492 nm wavelength and determined using a standard curve generated from hemolysed whole blood from the same mouse, taken as a terminal sample.

### Statistical Analysis

A *p* value < 0.05 was considered significant throughout this study. Statistical analysis was performed using computer software (InStat, Version 3.06, GraphPad Software, San Diego, CA, USA). Comparisons between two data sets were made using Student’s paired test. For multiple comparisons, one way Analysis of Variation (ANOVA) was employed, with post-tests. Normally distributed data sets with similar standard deviations were assessed with Tukey’s post-test, while data sets failing either criterion were assessed using non-parametric (Kruskal-Wallace) ANOVA with Dunn’s post-test. Data sets were logarithmically transformed if this transformation allowed them to meet the two criteria above.

## Results

### Recombinant protein design and expression

**Fig. 2:**
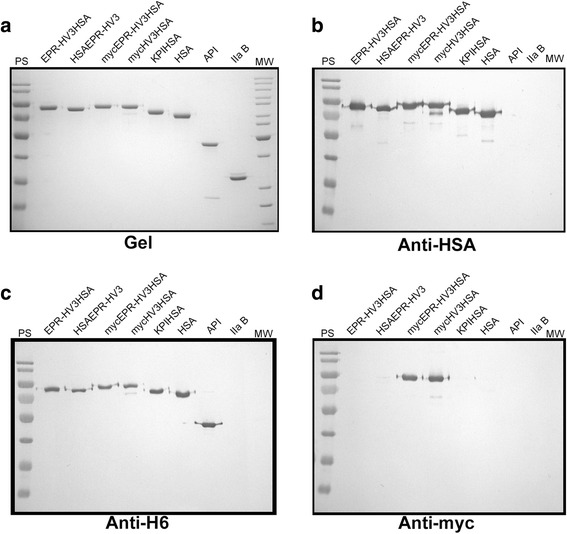
Electrophoretic and immunological characterization of fusion proteins.Panel **a** depicts a 10% SDS-polyacrylamide gel electrophoresed under reducing conditions and stained with Coomassie Brilliant Blue (Gel). Lanes contain markers (MW) or pre-stained markers (PS) or 500 ng of purified proteins (identified above the lanes). Recombinant His-tagged Alpha-1 Protease Inhibitor (API) (purified from transformed *E. coli* Top10 as described [39]), or human plasma-derived α-thrombin B chain (IIa B) served as non-HSA-related or non-HSA, non-His tagged controls. Panels **b**-**d** depict immunoblots of replicate gels identical to that shown in Panel **a**, except that 250 ng of purified proteins were used per lane, and gels were transferred to nitrocellulose and probed with antibodies specific to HSA, the hexahistidine tag, or the myc epitope (Anti-HSA, Anti-H6, or Anti-myc respectively). PS markers were (in kDa): 180; 130; 95; 72; 55; 43; 34; and 26. MW markers were (in kDa): 200; 150; 120; 100; 85; 70; 60; 50 (greater intensity); 40; 30; 25; and 20

Novel albumin fusion proteins were designed to test the effects of transient blockade of hirudin activity as shown in Fig. 1. The N-terminus of HV3 was blocked using a variety of different groups including: the FXIa-cleavable tripeptide EPR (in EPR-HV3HSA); its extended triskaidecapeptide counterpart mycEPR (in mycEPR-HV3HSA); the entire coding sequence of HSA (in HSAEPR-HV3); and noncleavable control decapeptide myc (in mycHV3HSA). Other proteins depicted in Fig. 1 have been previously described, including KPIHSA [22], a direct FXIa inhibitor-albumin fusion, His-tagged unfused HSA [14], and constitutively active HV3HSA (also called HV3HSAH_6_) [21, 30]. All proteins were expressed at similar levels and were purified from conditioned *P. pastoris* media; as shown in Fig. 2a, they demonstrated relative electrophoretic mobilities consistent with their predicted length in amino acids, given that aberrant migration of HV3-containing polypeptides relative to molecular mass standards has been previously noted and arises from clustered negative charges in the HV3 C-terminal dodecapeptide [14, 20, 21]. Immunoblotting revealed that all *P. pastoris*-derived fusion proteins reacted with anti-HSA (Fig. 2b) and anti-hexahistidine monospecific polyclonal antibodies (Fig. 2c), while only mycEPR-HV3HSA and mycHV3HSA reacted with anti-myc monoclonal antibodies (Fig. 2d). Immunoblotting results were supported by immunoreactivity with positive controls (e.g. anti-H6 antibodies reacted with recombinant API but not plasma-derived thrombin B chain, Fig. 2c).

### Initial screening of FXIa-activatable albumin fusion proteins

**Fig. 3:**
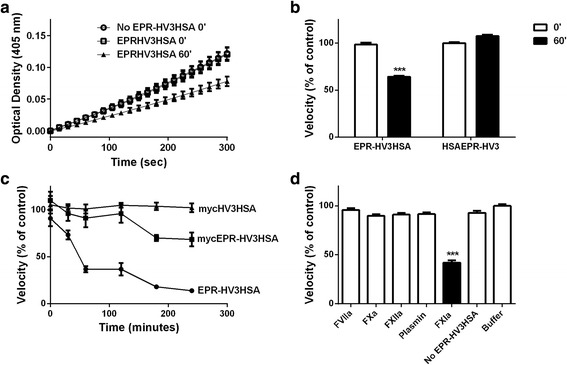
Activation of latent antithrombin activity in fusion proteins. Purified albumin fusion protein preparations were first reacted with specific purified proteases, then diluted into the presence of thrombin and its chromogenic substrate S2238, and the velocity of the thrombin-catalysed amidolysis reaction was measured and expressed as a percentage of the uninhibited control reaction. Panel A: EPR-HV3HSA was reacted with FXIa for zero (open squares) or 60 min (closed triangles) prior to dilution into the S2238 reaction and measurement of the rate of coloured product formation by thrombin over time. Buffer controls introduced phosphate buffer PPNE (see Materials and Methods) and FXIa (open circles) but no fusion protein into the S2238 reaction**.** Data represents the mean ± SEM, *n* = 7. Panel **b**: As in Panel **a**, EPR-HV3HSA or HSAEPR-HV3 proteins were incubated for zero (0′, white bars) or 60 min (60′, black bars) prior to dilution into S2238 reactions and determination of the rate of thrombin-mediated amidolysis. EPR-HV3HSA reactions are the slope of the same progress curves shown in Panel **a** as a percentage of the Buffer control; HSAEPR-HV3 reactions were determined analogously. Panel **c**: The mean (*n* = 6 ± SEM) of thrombin reaction velocities supplemented with activation reactions of the three fusion proteins labelled to the right of the progress curves with FXIa is shown, at six time points, in PPNE buffer as in Panels **a** and **b**. Panel **d**: As in Panel **a** and **b**, except that HBS / 5 mM CaCl_2_ was used as the buffer instead of PPNE and except that EPR-HV3HSA was combined with the proteases identified on the x axis prior to determination of second stage thrombin-mediated reaction velocity (white bars, FVIIa, FXa, FXIIa, or Plasmin; black bars FXIa). Control reaction “FXIa + No EPR-HV3HSA” contained FXIa but no EPR-HV3HSA in the activation reaction. Buffer reaction contained only HBS / 5 mM CaCl_2_ Data represents the mean ± SEM, *n* = 7

We sought an FXIa-activatable, slowly cleared form of hirudin. As in our previous work with plasmin-activatable hirudin and hirudin-albumin fusion proteins, we selected hirudin variant 3 (HV3), one of the most potent known variants of hirudin [21, 30]. We chose to assess all activatable and non-activatable forms of HV3 as albumin fusion proteins, to ensure slow clearance and an extended time of pharmacodynamic action. To assess if FXIa recognized the EPR-I cleavage site differently if it was positioned N- or C-terminal to HSA, we compared EPR-HV3HSA and HSAEPR-HV3 with respect to FXIa reactivity. We employed a two-stage assay, first challenging the EPR or HSAEPR blocking groups with FXIa, then diluting the first reaction products into a thrombin S2238 chromogenic activity assay. That FXIa in the first stage of the reaction had no effect on S2238 amidolysis was shown by the lack of difference between its presence or absence on the thrombin-catalysed initial rates (see Fig. 3a) and also by the lack of reaction of S2238 when FXIa was substituted for thrombin (data not shown). As shown in Fig. 3b, exposure of EPR-HV3HSA, but not HSAEPR-HV3, to FXIa conferred antithrombin activity on the first, but not the second preparation. We next compared two small N-terminal HV3 blocking groups, tripeptide EPR and triskaidecapeptide mycEPR, to determine if one was more readily recognized by FXIa than the other. Fig. 3c shows that FXIa preincubation did not activate control protein mycHV3HSA, and exposed greater antithrombin activity for EPR-HV3HSA than for mycEPR-HV3HSA (compare middle and lower progress curves in Fig. 3c). Activation appeared to be specific to FXIa, as it alone, among 5 coagulation or fibrinolytic enzymes that were tested, released antithrombin activity from EPR-HV3HSA (Fig. 3d; note that the buffer employed in this experiment differed from those shown in the other panels of Figure 3 to accommodate calcium sensitive proteases FVIIa and FXa). In each case, control experiments in which the protease (FVIIa, FXa, FXIIa, Plasmin, or FXIa) was introduced into the S2238 reaction without addition of albumin fusion proteins showed no difference compared to buffer only, activation protease-free results (data not shown). Accordingly, we selected EPR-HV3HSA over mycEPR-HV3HSA and HSAEPR-HV3 for further study.

### Antithrombotic activity of EPR-HV3HSA in the ferric chloride-treated murine carotid artery model

**Fig. 4:**
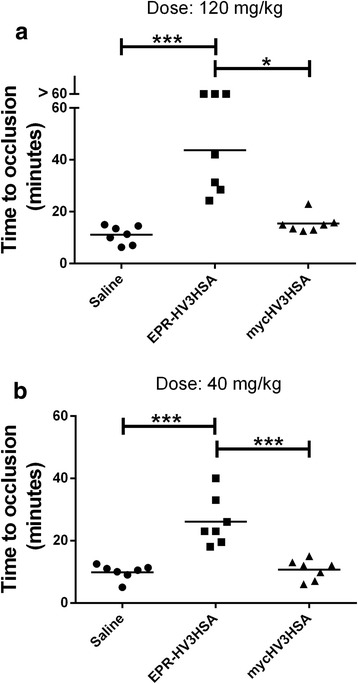
Time to occlusion of murine ferric chloride-treated carotid arteries. The time to occlusion (TTO) of surgically exposed carotid arteries treated by topical application of 10% (*w*/*v*ol) ferric chloride, for CD-1 mice receiving saline vehicle (circles) or the two albumin fusion proteins identified on the x axis, in saline (EPR-HV3HSA, squares, and mycHV3HSA, triangles). Mice were injected intravenously with protein doses of 120 mg/kg (Panel **a**) or 40 mg/kg (Panel **b**) two minutes prior to application of a ferric chloride-saturated patch (for three minutes). After patch removal blood flow through the artery was monitored for 60 min using ultrasound. Vessels not occluding during the observation period had their TTO recorded as 60 min. Each point in the scatter plots reflects results from a single mouse of a group of 7. Horizontal lines indicate the mean for each group. Lines above groups show statistical significance between groups as determined by ANOVA with post-tests (*p* < 0.5, *, *p* < 0.01, **, *p* < 0.001, ***)

FXIa-activatable EPR-HV3HSA and its non-activatable counterpart, mycHV3HSA, were compared in the ferric chloride-treated thrombosis model in the topically treated murine carotid artery, at two doses. Fig. 4 shows that EPR-HV3HSA administration prior to ferric chloride exposure lengthened the TTO of the artery four-fold versus vehicle, to 44 ± 16 min at 120 mg/kg body weight, and 2.6-fold versus vehicle, to 22 ± 3 min at 40 mg/kg body weight. In contrast, mycHV3HSA administration elicited TTO values that did not differ statistically from saline vehicle at both doses. We selected the lower dose of 40 mg/kg for further experimentation.

### Antithrombotic activity of EPR-HV3HSA in a mouse model of inflammatory thrombosis

**Fig. 5:**
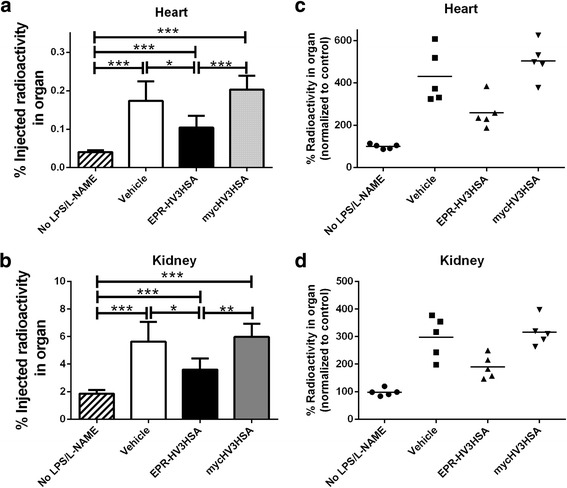
Deposition of ^**125**^I-fibrin(ogen) in organs of mice treated with LPS and L-NAME. Mice were treated with or without (No LPS/L-NAME) multiple intraperitoneal (IP) injections of LPS and L-NAME. Immediately prior to the IP injection, all mice were injected intravenously with ^**125**^I-fibrinogen in saline vehicle, alone (Vehicle) or combined with 40 mg/kg body weight EPR-HV3HSA or mycHV3HSA. Five hours later, mice were euthanized and the radioactivity present in excised hearts (panels **a** and **c**) or kidneys (panels **b** and **d**) was determined and expressed as the % of the injected dose (panels **a** and **b**) or normalized to the mean of the % of the injected dose in the No LPS/L-NAME cohort (panels **c** and **d**). Bars depict the mean of 5 determinations, each in a separate mouse, ± SD. Lines above bars show statistical significance between groups as determined by ANOVA with post-tests (*p* < 0.5, *, *p* < 0.01, **, *p* < 0.001, ***). Scatter plots (**c** and **d**) show the same data as in **a** and **b**, except that each point corresponds to data from a single mouse, normalized to the “No LPS/L-NAME” control

We next examined the ability of EPR-HV3HSA to modulate thrombosis in a murine endotoxemic model, in which co-administration of bacterial LPS and the nitric oxide antagonist L-NAME promotes fibrin deposition in organs [23]. We monitored ^125^I-labeled fibrin(ogen) in the heart and kidneys of treated mice. Mice treated with saline vehicle just prior to initiation of LPS/L-NAME demonstrated a significant, 4.3-fold elevation in heart fibrin(ogen) relative to mice not receiving LPS/L-NAME, one that was unaltered by administration of mycHV3HSA, but was significantly reduced, by on average 40%, by dosing with EPR-HV3HSA (Fig. 5a and c). The same pattern of results was observed in the kidneys of the same cohorts of mice; the 3-fold elevation in fibrin(ogen) deposition elicited by LPS/L-NAME administration was significantly, albeit partially, reduced, by on average 37%, by treatment with EPR-HV3HSA but not with mycHV3HSA (Fig. 5b and d). In contrast, treatment with 1.0 mg/kg KPIHSA, a fusion protein that inhibits FXIa with inhibitory constants in the low nM range [22], did not affect the LPS/L-NAME-mediated increases in fibrin(ogen) deposition(data not shown).

### Effects of EPR-HV3HSA in murine bleeding models

**Fig. 6:**
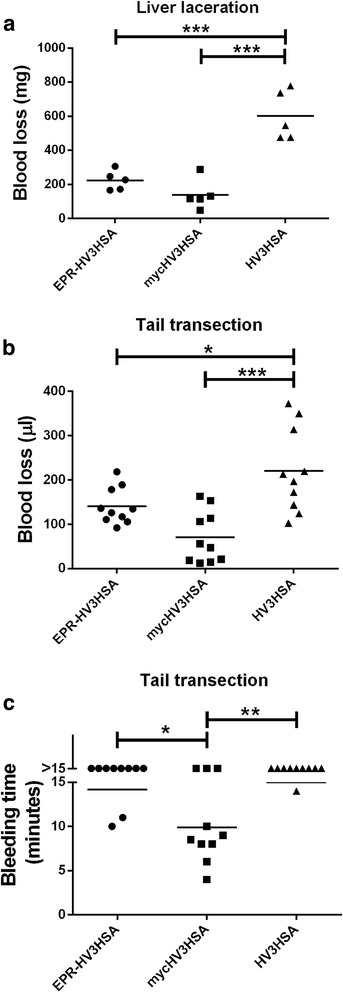
Blood losses or bleeding time in mice subjected to liver laceration or tail transection. Mice were subjected to liver laceration (Panel **a**) or tail transection protocols (Panels **b** and **c**). Blood losses were measured by weighing shed and/or clotted blood (**a**) or collecting shed blood into water and quantifying hemolysis (**b**). Continuing bleeding from the transected tail was assessed visually every 30 s for 15 min (**c**). Mice were injected intravenously with 40 mg/kg body weight of the purified proteins identified on the x axis of each panel. Scatter plots show each data point from a separate mouse in groups of 5 (panel **a**) or 10 (panels **b** and **c**) mice. Horizontal lines depict the mean for each group. Lines above bars show statistical significance between groups as determined by ANOVA with post-tests (*p* < 0.5, *, *p* < 0.01, **, *p* < 0.001, ***)

Having demonstrated antithrombotic activity of EPR-HV3HSA in two murine models of thrombosis, we next addressed the issue of whether EPR-HV3HSA promoted bleeding. In the liver laceration model, administration of constitutively active HV3HSA just prior to injury resulted in severe bleeding approaching 50% of the murine blood volume (600 ± 150 mg). In contrast, administration of EPR-HV3HSA or mycHV3HSA was associated with statistically indistinguishable post-injury blood losses of 220 ± 60 mg and 140 ± 90 mg, respectively (Fig. 6a). Similar results were obtained in the tail transection model, which constitutes a less severe hemorrhagic insult; EPR-HV3HSA was associated with significantly lower blood losses than HV3HSA (130 ± 40 μl versus 230 ± 100 μl) which did not differ significantly from those associated with mycHV3HSA administration (54 ± 40 μl) (Fig. 6b). In the same mice, no significant difference between bleeding times from the transected tail, as opposed to volumes of shed blood from that site, was found between mice treated with HV3HSA or EPR-HV3HSA, while mice treated with mycHV3HSA ceased to bleed significantly earlier than the other two groups (Fig. 6c). The proportion of mice still bleeding at the end of the observation period was 8/10 and 9/10 for the EPR-HV3HSA and HV3HSA groups, respectively, compared to only 3/10 for the mycHV3HSA-treated group (Fig. 6c).

### Stability of EPR-HV3HSA in human and murine plasma

**Fig. 7:**
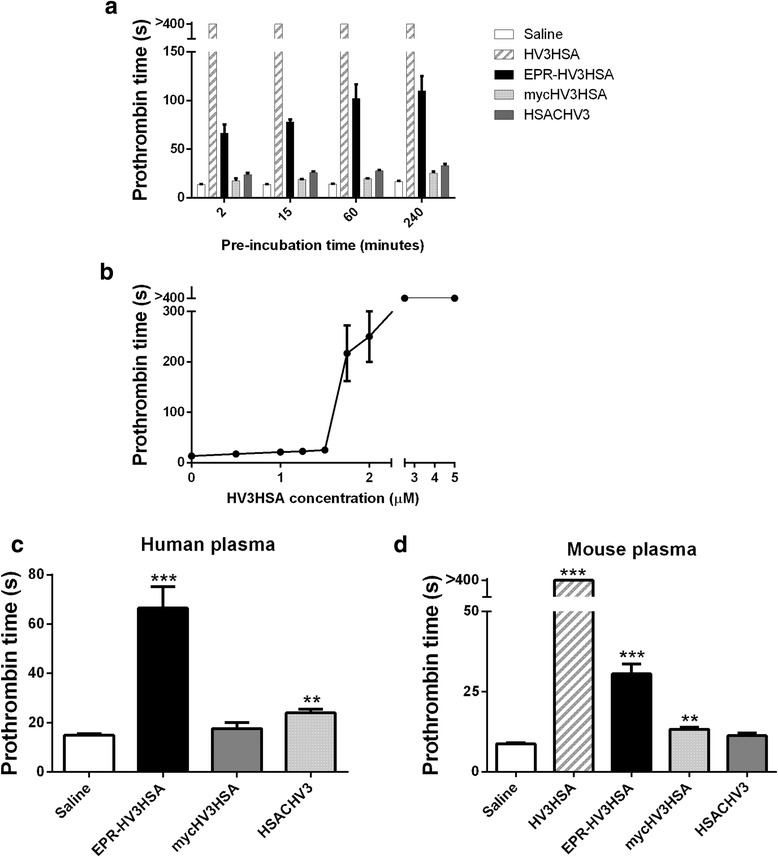
Stability of albumin fusion proteins in plasma**.** Panel **a**: Prothrombin times (PT) were measured for reactions containing 13.3 μM fusion protein in saline vehicle, or saline alone. Test samples were combined with human normal pooled plasma (NHPP) and preincubated at 37 °C prior to addition of calcium and PT reagent containing tissue factor for times indicated on the x axis. The mean of 12 determinations ± SD is shown for reactions containing test proteins or solutions shown in the legend at right, Panel **b**: As in Panel **a**, but for varying concentrations of purified HV3HSA preincubated with NHPP prior to PT initiation for two minutes. Data points are the mean of seven determinations ± SEM. Note y axis break. Panel **c**: As in Panel **b**, but for reactions supplemented with the proteins or solutions specified below the x axis. ***, *p* < 0.001 versus Saline by non-parametric ANOVA with Dunn’s post-tests. Panel **d**: As in Panel **c**, but mouse pooled plasma was substituted for NHPP. Note y axis break. **, *p* < 0.01, and ***, *p* < 0.001 versus Saline by non-parametric ANOVA with Dunn’s post-tests. Note that results of > 400 s mean that no clotting was detected at the end of the observation period

To gain additional insights as to the potential fate of injected EPR-HV3HSA in vivo, we examined its stability in plasma. Using normal human pooled plasma, we compared the PT in the presence of 13.3 μM albumin fusion proteins (the theoretical peak plasma concentration of the fusion proteins following a 1 mg/kg body weight intravenous dose to mice). As shown in Fig. 7a, plasma samples supplemented with HV3HSA failed to clot during the 400 s observation period in the assay. Mean PTs for plasma samples supplemented with saline, mycHV3HSA, or HSACHV3 were below 25 s, but the corresponding EPR-HV3HSA-containing samples exhibited PT values in excess of 60 s. A concentration curve of HV3HSA PT times after two minutes’ preincubation of chelated NHPP with the fusion protein (Fig. 7b) illustrated that HV3HSA had only a modest effect on the PT until concentrations in excess of 1.5 μM were exceeded; plasma supplemented with 2.5 μM HV3HSA failed to clot. By extrapolation, this finding suggests that 1.5 to 1.75 μM of the total 13.3 μM EPR-HV3HSA became activated within two minutes of exposure to calcium-chelated human plasma, or 11-13% of the administered dose. Fig. 7c shows PT values for saline and albumin fusion proteins (excepting HV3HSA, to enhance visibility in the < 80 s PT range). Inclusion of EPR-HV3HSA significantly prolonged the PT by 4-fold, while mycHV3HSA had no significant effect. A modest but significant elevation resulting from HSACHV3 inclusion in the PT was also observed. When we substituted mouse normal pooled plasma for NHPP in PT assays (Fig. 7d), similar results were observed. Inclusion of 13.3 μM HV3HSA in murine plasma PT assays eliminated clotting, while EPR-HV3HSA significantly increased the PT by 3- to 4- fold, while HSACHV3-containing assays did not differ from saline controls. The 6-fold prolongation in human plasma PT arising from inclusion of 13.3 μM EPR-HV3HSA was retained when pooled plasma depleted of Factor VIII, Factor IX, Factor XI, or Factor XII were substituted for NHPP (5- to 7- fold observed prolongation, data not shown).

## Discussion

Our initial preferred strategy in this study was to modify the previously described fusion protein HSACHV3 as little as possible, simply substituting a FXIa cleavage site for the plasmin cleavage site GSGIYR- that we had previously positioned between HSA and HV3 [14]. This geometry would have recreated the hypothetical situation whereby HSACHV3, encountering an activating enzyme in the local environment of a thrombus, released HV3 not only from inhibition by the polypeptide blocking its N-terminus, HSAC, but also physically released HV3 from union with albumin, restoring rapid clearance of the small protein. The only difference would have been substitution of FXIa for plasmin as the putative thrombus-localized activator. However, we found that positioning the EPR cleavage site between HSA and HV3 in fusion protein HSAEPR-HV3 prevented its recognition by FXIa. In contrast, the alternative geometry, represented by fusion protein EPR-HV3HSA, was recognized by FXIa, at least to the extent that detectable antithrombin activity could be liberated in vitro. For in vivo applications, however, we were aware that EPR-HV3HSA activation would liberate HV3HSA, a constitutively active hirudin-albumin fusion protein that would perpetuate or conceivably worsen the narrow therapeutic window of hirudin [31]. Our ability to activate EPR-HV3HSA was entirely consistent with the findings of Zhang et al., who reported that hirudin variant 2 (HV2) activity was liberated by FXIa cleavage of recombinant protein EH, in which EPR was positioned on the N-terminus of HV2 [15]. These investigators also noted no activation of EH by FXa or thrombin; we extended this finding to include a lack of reactivity with plasmin, FVIIa, FXa, or FXIIa, supporting the specificity of the EPR hirudin “switch”.

EPR is nevertheless a very small blocking group, comprising a mere three amino acid residues highly exposed on the N-terminus of both EPR-HV3HSA and EH. To provide additional insulation of the HV3 domain from plasma, we produced fusion protein mycEPR-HV3HSA, expanding the blocking group to 13 amino acids. However, this protein was recognized less effectively by FXIa than EPR-HV3HSA; under conditions in which detectable antithrombin activity was liberated from EPR-HV3HSA in 20 min, 200 min of digestion was required to liberate similar amounts of functional HV3HSA from mycEPR-HV3. We cannot exclude the possibility that another blocking group of the same size, perhaps one with a lesser density of negatively charged residues than the myc epitope (sequence EQKLISEEDL) might be better recognized by FXIa.

The FXIa-dependent activation of HV3HSA that we observed in vitro was similar to that we previously observed for HSACHV3 by plasmin [14] in that high nM concentrations and minutes of incubation were required to generate measurable release of active HV3HSA or HV3. Both for plasmin and for factor XIa, it is difficult to predict what local concentrations might be generated in a thrombus. Free plasmin is generally not detectable in plasma, in part due to the efficiency with which it is incorporated into plasmin-antiplasmin complexes [32]; and free FXIa has been reported to be present in picomolar concentrations in the plasma of healthy individuals [33]. Plasma concentration data is not necessarily predictive of local concentrations that could be generated in a thrombus. In the case of HSACHV3, it was possible to show a favourable profile of antithrombotic activity with minimal bleeding risk when in vitro studies were extended into murine in vivo studies [19]; given this precedent, we decided to examine EPR-HV3HSA activities in vivo, even though the plasmin precursor plasminogen is present in approximately 20-fold excess over factor XI in plasma [34].

Fusion protein EPR-HV3HSA showed antithrombotic activity in two models of thrombosis in mice, one dependent on ferric chloride, and one independent of this agent. Ferric chloride, although widely used as a prothrombotic treatment, is a potent antioxidant with pleiotropic effects, and it has been suggested that its use should be accompanied in rigorous experimental studies with data from another model [35]. EPR-HV3HSA, at 120 mg/kg or 40 mg/kg, prolonged the TTO in the ferric chloride-treated carotid artery, while mycHV3HSA had no greater effect than saline vehicle. These results are consistent with the findings of Zhang et al. [15], who noted a significant increase in the TTO in a rat carotid artery model in which thrombosis was initiated by application of an electrical current, and animals were treated with 4 mg/kg body weight EH, an equimolar dose to the larger EPR-HV3HSA protein at 40 mg/kg. In the LPS/L-NAME model, in which more physiologically relevant thrombotic triggers of inflammation and nitric oxide antagonism are employed, EPR-HV3HSA reduced fibrin deposition in the heart and kidneys. Interestingly, a direct FXIa inhibitor, KPI, also administered as an albumin fusion protein at an identical dose by weight, was without effect. This finding suggests that although FXIa is generated in the LPS/L-NAME model, its inhibition is insufficient to prevent thrombosis, while its use to trigger HV3 liberation and thrombin inhibition was effective. A possible explanation of this effect is that the affinity of KPI for FXIa is much less than that of HV3 for thrombin, with binding constants for the former reported in the nM range [36] and for the latter interaction in the sub-pM range [31], a difference of more than three orders of magnitude. While EPR-HV3HSA reduced intra-organ fibrin deposition in the LPS/L-NAME setting, it did not eliminate it; in this regard it was less effective than recombinant activated protein C in our previous use of this model [23].

EPR-HV3HSA clearly promoted bleeding to a lesser extent than constitutively active HV3HSA, as shown by significantly lower blood losses than those elicited by that protein in both liver laceration and tail transection models. However, mean blood losses were greater in EPR-HV3HSA-treated mice than in mycHV3HSA-treated mice in both models, although the differences did not reach statistical significance. Bleeding times in the tail transection model were significantly lower in mycHV3HSA-treated mice than in HV3HSA- or EPR-HV3HSA-treated mice; therefore this bleeding parameter was not reduced versus constitutively active HV3HSA. In our previous studies with HSACHV3, blood losses were more convincingly reduced to baseline in both bleeding models than in the current study with EPR-HV3HSA [19]. These observations could reflect the relatively small number of mice that we tested, or they could indicate that FXIa was a less appropriate trigger for HV3 liberation than plasmin, or they could point to instability of the small EPR blocking group in plasma. To distinguish among these possibilities, we examined the stability of EPR-HV3HSA and, to a lesser extent, that of HSACHV3 in plasma.

At a fusion protein concentration equivalent to the peak concentration expected in the murine circulation following intravenous injection of 40 mg/kg body weight doses, HV3HSA eliminated the ability of human or murine plasma to clot in the PT assay, while HSACHV3 had no effect in murine plasma and a modest, less than 2-fold prolongation in human plasma. In contrast, EPR-HV3HSA, after only two minutes’ exposure to chelated plasma, prolonged the PT by five-fold in human plasma, and four-fold in mouse plasma. Prolonging the incubation in plasma increased the PT, but loss of clotting factor activities over time would also be expected to contribute to such an effect. Comparison to a calibration curve with HV3HSA suggested that approximately 10 – 15% of EPR-HV3HSA was behaving as if its EPR blocking group had been removed, in both species’ plasma. The trivial explanation that proteolysis had taken place during purification of EPR-HV3HSA could be eliminated because EPR-HV3HSA had no antithrombin activity without exposure to purified FXIa or plasma.

While we cannot eliminate all proteases specifically involved in coagulation or fibrinolysis in this unexpected activation, its detection in chelated plasma renders their involvement unlikely. Moreover, unexpected activation of EPR-HV3HSA was also noted in plasma depleted of Factor VIII, Factor IX, Factor XI, or Factor XII, and EPR-HV3HSA also showed no sensitivity to purified FVIIa or plasmin. A more likely explanation for these findings is that EPR-HV3HSA, at μM concentrations, lost its EPR blocking group through the action of plasma endopeptidases or exopeptidases unrelated to coagulation or fibrinolysis. Proteomic studies of human plasma have shown that 28% of 722 unique N-terminal blood protein peptides had characteristics consistent with their having arisen through aminopeptidase action, such as laddering [37]. In future experiments, this problem could conceivably be avoided by rendering EPR more accessible to FXIa in an internal location in a fusion protein like HSAEPR-HV3, either by separating EPR from HSA structures using an optimized polyglycine linker, or by using a different FXIa cleavage site (like TSKTLR in coagulation factor IX [38]), or both.

## Conclusions

Fusion protein EPR-HV3HSA’s latent antithrombin activity was released by FXIa in vitro to a greater extent than mycEPR-HV3HSA; moving the EPR FXIa cleavage site to the C-terminus of HSA, in HSAEPR-HV3, abrogated FXIa recognition. EPR-HV3HSA was recognized and activated in vivo in mice, as shown by its antithrombotic activity in carotid artery and intra-organ thrombosis models, and the lack of activity of noncleavable mycHV3HSA. EPR-HV3HSA elicited less blood loss than constitutively active HV3HSA in lacerated liver and transected tail mouse models, but failed to attenuate the elevated bleeding time associated with HV3HSA administration. The partial overlap in bleeding promotion with HV3HSA is likely due to the partial instability of EPR-HV3HSA in murine and human plasma. Activation of EPR-HV3HSA in plasma was not due to any of seven tested coagulation or fibrinolytic proteases, but may have arisen due to the action of plasma exopeptidases on the N-terminally exposed EPR tripeptide. Only modest activation of plasmin-activatable HSACHV3 was observed. Although our results supported the use of FXIa as a thrombosis-specific fusion protein activator, N-terminally positioned EPR is a suboptimal biochemical “switch” compared to the internal plasmin-dependent switch in HSACHV3.
